# Unraveling Obesity: Transgenerational Inheritance, Treatment Side Effects, Flavonoids, Mechanisms, Microbiota, Redox Balance, and Bioavailability—A Narrative Review

**DOI:** 10.3390/antiox12081549

**Published:** 2023-08-03

**Authors:** Ruth Naomi, Soo Huat Teoh, Shariff Halim, Hashim Embong, Zubaidah Hasain, Hasnah Bahari, Jaya Kumar

**Affiliations:** 1 Department of Human Anatomy, Faculty of Medicine and Health Sciences, Universiti Putra Malaysia, Serdang 43400, Malaysia; gs60018@student.upm.edu.my; 2 Advanced Medical and Dental Institute, Universiti Sains Malaysia, Kepala Batas 13200, Malaysia; soohuat@usm.my; 3 Faculty of Health Sciences, University Technology Mara (UiTM) Pulau Pinang, Bertam Campus, Kepala Batas 13200, Malaysia; halimshariff@uitm.edu.my; 4 Department of Emergency Medicine, Faculty of Medicine, Universiti Kebangsaan Malaysia, Kuala Lumpur 56000, Malaysia; hashimembong77@ukm.edu.my; 5 Unit of Physiology, Faculty of Medicine and Defence Health, Universiti Pertahanan Nasional Malaysia, Kuala Lumpur 57000, Malaysia; 6 Department of Physiology, Faculty of Medicine, Universiti Kebangsaan Malaysia, Kuala Lumpur 56000, Malaysia

**Keywords:** obesity, natural antioxidants, phytochemical, oxidative stress, signaling mechanism

## Abstract

Obesity is known as a transgenerational vicious cycle and has become a global burden due to its unavoidable complications. Modern approaches to obesity management often involve the use of pharmaceutical drugs and surgeries that have been associated with negative side effects. In contrast, natural antioxidants, such as flavonoids, have emerged as a promising alternative due to their potential health benefits and minimal side effects. Thus, this narrative review explores the potential protective role of flavonoids as a natural antioxidant in managing obesity. To identify recent in vivo studies on the efficiency of flavonoids in managing obesity, a comprehensive search was conducted on Wiley Online Library, Scopus, Nature, and ScienceDirect. The search was limited to the past 10 years; from the search, we identified 31 articles to be further reviewed. Based on the reviewed articles, we concluded that flavonoids offer novel therapeutic strategies for preventing obesity and its associated co-morbidities. This is because the appropriate dosage of flavonoid compounds is able to reduce adipose tissue mass, the formation of intracellular free radicals, enhance endogenous antioxidant defences, modulate the redox balance, and reduce inflammatory signalling pathways. Thus, this review provides an insight into the domain of a natural product therapeutic approach for managing obesity and recapitulates the transgenerational inheritance of obesity, the current available treatments to manage obesity and its side effects, flavonoids and their sources, the molecular mechanism involved, the modulation of gut microbiota in obesity, redox balance, and the bioavailability of flavonoids. In toto, although flavonoids show promising positive outcome in managing obesity, a more comprehensive understanding of the molecular mechanisms responsible for the advantageous impacts of flavonoids—achieved through translation to clinical trials—would provide a novel approach to inculcating flavonoids in managing obesity in the future as this review is limited to animal studies.

## 1. Introduction

Obesity is defined as a body mass index (BMI) of 30 kg/m^2^ or higher. Increased neonatal morbidity and mortality are among the common notable consequences of obesity [1]. Globally, at least 10–30% subjects are reported to be obese [2]. An increased risk of metabolic dysfunction, lipotoxicity, fetal overgrowth, and an impaired neurodevelopmental process in F1 generation are some other common negative impacts of obesity [1]. In addition, the normal vascular structure of the blood vessels is altered in obese subjects. Resultantly, an increased level of stiffness, permeability, and diameter of the vessels is common in obesity, which may lead to thromboembolic complications [3].

There is increasing evidence that shows that obesity contributes to the inheritance of obesity among children; this means that pregnant mothers can pass on their epigenome to their offspring by transferring the parent cell’s germline to their child. This will eventually modify the epigenome of the embryo stem, causing obesity development in their child sooner or later [4]. In obesity, hypertrophy and hyperplasia are common clinical pathologies. Specifically, in maternal obesity, this may increase the production of pro-inflammatory factors such as tumor necrosis factor (TNF)-α, interleukin (IL)-6, and adipokine hormones. Dysregulation in the release of inflammatory factors will enhance the oversupply of nutrients to the fetus, leading to fetus overgrowth [5], thereby increasing the risk of developing metabolic diseases in their later life [6].

In addition, obesity is closely associated with an increased risk of neurodevelopmental delay, which worsens problem solving skills and inattentiveness [7]. Studies on an animal model fed a high fat diet (HFD) have shown a serious consequence of impaired neural circuits [8]. Numerous in vivo studies have further proven the link between obesity and cognitive delay, involving the underlying biological mechanism related to insulin resistance, leptin, increased fat mobilization, brain-derived neurotrophic factor (BDNF), and cytokines [7]. Obesity is proven to be a transgenerational vicious cycle and the current available treatments are often accompanied by adverse effects. Thus, natural derivatives, such as flavonoids, are said to be an effective method in managing obesity.

## 2. Transgenerational Inheritance of Obesity

Obesity is a transgenerational vicious cycle, which means that obesity can be passed from maternal and paternal parents to the child [9], and can last up to three generations in a family tree [10]. One of the most common phenomena in obesity is maternal overnutrition to the growing fetus, especially in cases of the excessive consumption of a high fat diet (HFD) [11]. Maternal overnutrition could induce epigenome modifications in the fetal programming, thereby resulting in the methylation of deoxyribonucleic acid (DNA), histone modifications, and the expression of non-coding RNA [12], which is linked with genomic imprinting [13]. This imprinting is a permanent programming that cannot be reversed or reprogrammed. As a result, it facilitates the transmission of defective genes to the offspring [14]. DNA methylation can alter the expression of obesity-associated genes, such as neuropeptide Y (NPY) [15], peroxisome proliferator-activated receptor gamma (PPARγ) [16], fatty acid binding protein 4 (FABP4) [17], and sterol regulatory element-binding protein-1 (SREBP-1) [18]. DNA methylation is a process that involves adding a methyl group to a cytosine residue in a CpG dinucleotide sequence, resulting in the formation of 5-methylcytosine. During organogenesis and tissue differentiation, maternal overnutrition triggers DNA methylation in the fetus through one-carbon metabolism. In this process, the addition of methyl groups to support all methylation reactions is reliant on the intake of dietary methyl donors and cofactors. This can lead to changes in the epigenetic pattern in the fetus [6]. The hypermethylation of the europeptide Y (NPY) and proopiomelanocortin (POMC) genes in the hypothalamic circuits may result in an increase in the NPY levels within the central amygdala. This, in turn, can lead to hyperphagia, or increased food intake [19], while the action of the peripheral signals—such as leptin and insulin, which contribute to the feeling of satiety—can be impeded by the overexpression of POMC in the arcuate nucleus [20].

Similarly, maternal overnutrition may cause defects in the PPARγ by enhancing the overexpression of PPARγ in the placenta [21]. As PPARγ is vital for the formation of the labyrinthine layer of the placenta and for the modulation of the genes that are responsible for the production of sarcoplasmic and adipose tissue [22], the increment of PPARγ enhances adipogenesis [6]. On the other hand, PPARγ binds to peroxisome proliferator response element (PPRE) in the promoter regions of the target genes involved in glucose and lipid metabolism, and activates their expression [23]. In this way, the activation of PPARγ can regulate lipid metabolism-related genes, including HSL and ATGL, which can affect lipid breakdown and adipocyte differentiation [24]. Similarly, the binding of PPARγ to insulin receptor substrate 1 (IRS1) [25] and glucose transporters (GLUT) 1 and 4 may repress the activity of IRS1 [25] and GLUTs 1 and 4 [26], thereby causing an imbalance in glucose homeostasis by increasing the uptake of glucose. Both of these scenarios may result in excessive weight gain.

**Figure 1:**
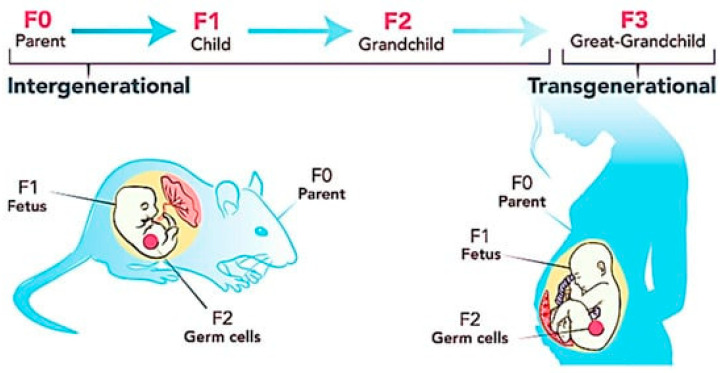
Transgenerational inheritance pattern of obesity. Figure reused under the permission granted by http://creativecommons.org/licenses/by/4.0/ (accessed on 23 March 2023) [15].

In addition, histone modification is one of the factors that can cause obesity inheritance. Histone modifications and DNA methylation are overlapping processes. Maternal overnutrition can suppress histone acetylation and enhance histone methylation. During the process of histone acetylation, transcription factors attach to genes, which stimulates an increase in their expression, facilitated by the histone acetyltransferase enzyme. The suppression of histone acetylation is positively associated with a decrease in the gene expression that is responsible for fat metabolism. For example, histone H4 acetylation in the offspring’s hippocampus can inhibit the activity of genes [6] encoding acyl-CoA synthetase long-chain family member 1 (ACSL1) [27], carnitine palmitoyltransferase 1 (CPT1) [28], and acyl-CoA dehydrogenase (ACADM) [29] in the adipocytes. As these genes are involved in the breakdown and utilization of fatty acids, the decreased expression of these genes may impair fat metabolism, thereby enhancing fat deposition [27,28,29]. In addition, the expression of non-coding RNA, including microRNAs, is a closely linked epigenetic mechanism associated with the inheritance of obesity. The maternal overnutrition-induced overexpression of non-coding RNA can cause modifications in microRNAs, resulting in impaired lipid metabolism and insulin resistance in offspring. Consequently, during weaning, the offspring tend to have an increase in body weight and impaired glucose metabolism [6]. Figure 1 summarizes the transgenerational inheritance pattern of obesity.

## 3. Current Available Treatment and Its Side Effects

There are several methods said to be effective in managing obesity in the modern era. However, they are all often accompanied by adverse effects, especially if the medicines are over consumed, or in the case of exercises, a lack of supervision can result in muscle cramps or injuries. In fact, two of the most commonly prescribed medicines, Sibutramine and Lorcaserin, have been recalled by the FDA as anti-obesity medicines. This is mainly because sibutramine increases the risk of cardiovascular disease [30], while Lorcaserin is shown to increase the incidence of certain types of cancers [31]. Table 1 summarizes the current available interventions for managing obesity and their side effects.

## 4. Flavonoids and Their Sources

**Figure 2:**
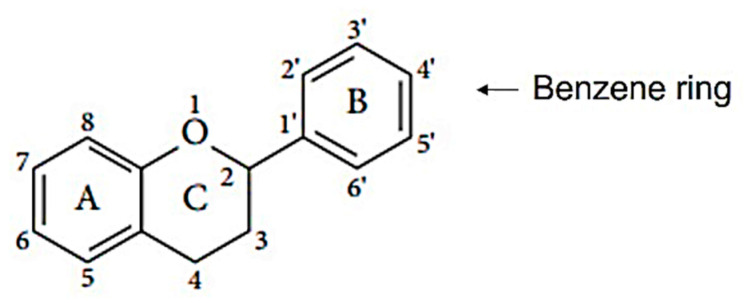
Basic structure of flavonoids.

Flavonoids are naturally occurring compounds that are found in growing plants. Along with carotenoids and chlorophylls, these pigments are widely distributed and found in almost all plants. Plant roots, stems, leaves, flowers, and fruits are reported to have a high concentration of flavonoids [45]. They are known for giving fruits, flowers, and seeds taste and fragrance [46]. Structurally, flavonoids are water-soluble compounds containing two benzene rings (A and B) with phenolic hydroxyl. It is a large class of compound based on the C_6_-C_3_-C_6_ skeleton, as shown in Figure 2.

Flavonoids are synthesized from the condensation of one molecule of phenylalanine with three molecules of malonyl-CoA. They are all structurally descended from parent compounds called flavones, which are typically discovered in the sap of immature plant tissues. This reaction yields chalcone, catalyzed by chalcone synthase, and is followed by chalcone isomerization, which leads to the formation of flavanone. Then, it produces various types of flavonoid groups [47,48]. In nature, flavonoids usually exist together with other compounds; when dietary foods are rich in flavonoids, they often take in other compounds, and flavonoids can also interact with other compounds, such as carbohydrate, fat, protein, acid, etc. The majority of flavonoids are found in nature as glycosides, which are water-soluble due to the presence of sugars and hydroxyl groups, whereas the presence of methyl groups and isopentyl units make flavonoids lipophilic. Flavonoids can be classified into several classes, including isomers of flavonoids and their hydrogenated reductions; namely, flavonols, flavanols, flavanones, flavones, isoflavones, and Anthocyanidins [45], as shown in Table 2.

The chemical structure–activity relationship of flavonoids can provide insights into how specific structural features influence their activity in managing obesity. Here are some key structural elements of flavonoids and their potential impact on their anti-obesity effects. The structure of flavonoids consists of two aromatic rings (i.e., A and B rings) that are connected by a 3-carbon chain, which forms an oxygenated heterocyclic C ring [49]. Typically, multiple hydroxyl groups (–OH) are attached to the phenolic rings of flavonoids. The number and positioning of these hydroxyl groups are important as they determine the antioxidant and anti-inflammatory activities of flavonoids, which are relevant to obesity management [50]. Higher numbers of hydroxyl groups generally correlate with increased antioxidant potential [50].

Substitutions on the A- and B-rings were found to affect their biological activities. For instance, hydroxylation at the 3′- and 4′-positions of the B-ring enhances the inhibitory effect of flavonoids on adipogenesis [51]. In addition, the B-ring’s catechol moiety (two adjacent hydroxyl groups) is linked to increased thermogenic activity and energy expenditure [52]. Flavonoids may have either a C-ring consisting of a benzene ring (C_6_) or a fused heterocyclic ring, such as pyran or pyrone (C_6_-C_3_). Previous studies found that flavonoids with a larger and saturated C-ring exert powerful inhibitory effects on adipocyte differentiation [52]. The A-ring of flavonoids can undergo various modifications, including glycosylation, methylation, and prenylation [51]. These substitutions can affect the bioavailability and metabolic stability of flavonoids. For example, the methylation of the hydroxyl groups in the A-ring may enhance their lipophilicity and cellular uptake [53]. Flavonoids with conjugated double bonds were shown to contribute to antioxidant and anti-inflammatory activities. Therefore, the positions of these double bonds can impact the stability and reactivity of flavonoids [51,52].

Nonetheless, the chemical structure–activity relationship of flavonoids is complex and varies depending on the specific compound and molecular target being studied. Moreover, other factors, such as stereochemistry, glycosylation patterns, and the overall molecular shape, may influence their activity.

**Table 2:** Classification of flavonoids.

Classification	Structure	Sources	Examples of Compounds
Flavonols [46,47]	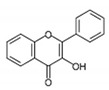	Apples, tea, wine, broccoli, black tea, olive oil	Kaempferol, quercetin, dihydroquercetin, prunetin, and rutin
Flavanols [46]	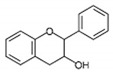	Coco, dark chocolate, blackberry, green tee, apple, wine, kiwi	Catechin, gallocatechin, and epicatechin,
Flavanones [46,54]	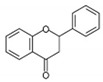	Citrus fruit, orange, tomato, lemon	Eriodictyol, naringenin, hesperetin, isosakuranetin, and their glycosides
Flavones [46,54,55]	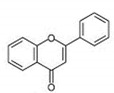	Legumes, cereal, angiosperms, mint, ginkgo biloba, thyme	Apingenin, Luteolin flavone, and their glycosides
Isoflavones [46]	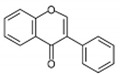	lentils, fava beans, soybeans	Genistein, daidzein, glycitein, equol, biochanin A, and formononetein
Anthocyanidins [56,57]	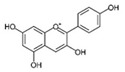	Berries, currants, grapes, blue-colored leafy vegetables, grains, cranberries	Malvidin, cyanidin, delphinidin, pelargonidin, and petunidin

## 5. Mechanism of Action of Flavonoids as a Natural Antioxidant in Managing Obesity

Flavonoids target numerous signalling pathways in managing obesity. One of the most common pathways that flavonoids target is the PPAR, as shown in Table 3. Flavonoids interact with and modify the activity of PPAR isotypes, such as PPARα, PPARβ/δ, and PPARγ [58,59,60], which play various roles and functions in metabolism. While PPARα is abundantly present in the liver and controls lipid metabolism, PPARβ/δ is omnipresent and governs glucose and fatty acid metabolism [60,61]. In addition, PPARγ regulates adipocyte differentiation and lipid synthesis [58]. The PPAR is known as a transcription factor in which the nutritional signals are translated into a pattern of gene expression that govern cellular bioenergetics. In turn, these receptors act as nutritional sensors that modify the systemic metabolism by regulating the metabolic processes across various organs [62]. The gene expression modulated by the PPAR includes genes that are involved in lipid metabolism, glucose homeostasis, and inflammation in obesity. For instance, PPARγ regulates the gene expression responsible for adipocytes differentiation, lipidic signalling, glucose metabolism, and the storage of lipids [63]. PPARγ forms heterodimers with RXR; subsequently, the binding of PPARγ-RXR to PPREs in the promoter regions of the target genes activates the transcription of genes such as leptin [64], lipoprotein lipase, aquaporin 7, adiponectin [63], and GLUT-4 [65], thereby inducing the adipocytes’ differentiation and hypertrophy by stimulating the expression of adiponectin [66]. Among these, Apigenin is proven to bind directly with PPARγ, and suppressed its downstream modulators (NF-κB) in an obese animal model. In this case, the intraperitoneal injection of Apigenin for 21 days successfully inhibited the nuclear translocation of p65, causing the PPARγ/p65 complex to relocate, together with the polarization of macrophages. As a result, the phosphorylation of p65 is suppressed, thereby inactivating NF-κB [67]. Resultantly, the high amount of adipocytes-induced inflammatory signalling pathways in obesity is repressed [68]. Similarly, Apigenin inhibits adipocytes differentiation by stimulating 5′-adenosine monophosphate-activated protein kinase (AMPK) phosphorylation via the downregulation of the PPARγ pathway [69].

It has been reported that flavonoids have numerous modes of action, including the potential to reduce high body weight by elevating COX-2 activity [70]. COX-2 is involved in the synthesis of prostaglandins, which have a role in inflammation and adipogenesis [71]. Flavonoids modulate COX-2 activity, altering the prostaglandin levels and affecting the regulation of body weight [70]. Despite being known as an adipogenic gene, the reduced expression of COX-2 has been linked to the stimulation of white adipose tissue formation [71]. The reduced expression of COX-2 results in less PGE2 being produced, which in turn promotes PKA signaling, which increases adipocyte differentiation and the formation of white adipose tissue [71]. Flavonoids increase COX-2 activity, affecting the synthesis of prostaglandins, particularly PGE2, which may reverse high body weight by counteracting the decreased COX-2 levels associated with adipogenesis [72]. In addition, COX-2 suppresses the activity of migration inhibitory factor (MIF) in the adipocytes, which is a vital process in macrophage polarization to an M1 phenotype [73]. In obesity, the excessive level and expansion of adipocytes promote the release of MIF in the epididymal adipose tissue. The high level of MIF exacerbates the existing inflammatory processes and promotes the transition from a metabolic state to an inflammatory state [74]. However, baicalein enhances the level of COX-2, accompanied by a significant reduction in the level of metalloproteinase (MMP)-2 and triglycerides [75]. In fact, NF-κB also represents a family of inducible transcription factors that play a critical role in inflammatory reactions by promoting the production of proinflammatory proteins, including iNOS, cytokines, and COX-2, following exposure to stimuli such as cytokines, foreign pathogens, or general stressful insults [76]. In adipogenesis, it has been reported that NF-κB is activated, leading to an increase in the expression of COX-2. This upregulation of COX-2 expression is believed to be crucial for the development of adipocytes as it stimulates the generation of prostaglandins that encourage the differentiation of preadipocytes [77]. However, it is important to acknowledge that COX-2 activation has been demonstrated to be one of the primary causes of inflammation associated with obesity. It has been suggested that NF-κB activity in adipocytes is also elevated in obesity, resulting in increased COX-2 expression and enhanced prostaglandin production. The inflammation fostered by these prostaglandins can lead to insulin-resistance and other metabolic issues [78]. Comparably, the oral administration of 150 mg/kg Kaempferol for 10 weeks efficiently lowers the plasma triglycerides content via suppressing the activity of SREBP-1 [79]. SREBP-1 is a transcription factor that plays a vital role in stimulating the hepatic de novo lipogenesis [18]. The nuclear transcription of the N terminal fragment from SREBP-1c activates SREBP-1 [80], and this process is modulated by AKT/protein kinase B via multiple pathways. These pathways include the downregulation of insulin-induced gene-2, the direct phosphorylation of SREBP, or the inhibition of glycogen synthase kinase (GSK)-3β. All of these may eventually result in AKT/protein kinase-stimulated SREBP-1 nuclear translocation, which may enhance hepatic lipogenesis [79]. Even so, the administration of flavonoid compounds, such as myricetin and naringenin, has proven to suppress dyslipidemia and promote weight loss by interfering with the Akt pathway [81]. 

In addition, inhibiting the action of pancreatic lipase is another mechanism through which flavonoids exhibit their anti-obesity property. Pancreatic lipase is an enzyme that hydrolyses triglycerides into fatty acids and glycerol to form monoglycerides [82], which will be then absorbed into the enterocytes of the small intestine. This is followed by the re-esterification of monoglycerides into triglycerides in the endoplasmic reticulum via the monoacylglycerol pathway. The triglycerides are then packed into lipoprotein chylomicrons and will enter the blood circulation via the lymphatic system. From the circulation, the chylomicrons will enter various organs for storage [83]. In the small intestine, pancreatic lipase hydrolyzes dietary lipids into fatty acids and glycerol [84]. FAS plays an important role in lipogenesis through fatty acid synthesis from acetyl-CoA and malonyl-CoA [85], whereas ATGL mediates lipolysis by breaking down stored triglycerides into fatty acids and glycerol [86]. In the case of obesity, overnutrition causes an alteration in the hepatic fatty acid metabolism, over-stimulating the activity of the pancreatic lipase, thereby causing a high level of triglycerides to be accumulated within the non-adipose organs, such as the liver [87].

Furthermore, 5 mg/kg of intragastric administration of quercetin [88] and 50 mg/kg oral administration of galangin [89] effectively suppressed pancreatic lipase activity by reducing the FAS and stimulating the release of ATGL in adipocytes. In a separate study, apigenin, a flavonoid compound, was found to reduce TG accumulation only at the highest dose (25 µM); however, none of the doses of apigenin displayed anti-adipogenic activity [90]. The study also revealed reduced lipogenesis by apigenin through the reduced expression of the fatty acid synthase (FASN) gene, which is involved in lipogenesis, and the increased gene expression of the lipolysis-related adipose triglyceride lipase gene by apigenin. Consequently, the lipogenesis and adipogenesis processes are inhibited [90]. Another selective route for flavonoids in exerting anti-obesity could be via the cellular energy sensor, known as AMPK. As a result of the decrease in the ATP or comprised state of the cellular energy status (increased of AMP:ATP status), the AMPK pathway will become activated [91]. The activation of AMPK will inhibit the energy-consuming anabolic pathway, involving gluconeogenesis, glycogen, fatty acid, triglyceride, cholesterol, and protein synthesize via the mTOR-p70SK-E2 signalling mechanism. Simultaneously, the catabolic pathway involving fatty acid oxidation and glycolysis will become activated [92], thereby ameliorating obesity [93,94]. In this instance, anthocyanin-rich bilberry extract [95] and citrus flavonoids such as hesperidin, narirutin, nobiletin, sinensetin, and tangeretin activate AMPK in the adipocytes. In addition, citrus flavonoids stimulate the phosphorylation of GSK-3β and suppress the level of HMGCR, a primary enzyme involved in the biosynthesize of cholesterol [96].

In addition, flavonoid extract from citrus aurantium L. polymethoxyflavones (nobiletin, tangeretin and sinensetin) [97] and quercetin [98] promoted weight loss in an obesogenic animal model by activating the thermogenesis genes and mitochondrial biogenesis in the brown adipose tissue [97,98]. The initiation of thermogenesis genes in the adipocytes increases energy expenditure by enhancing the activity of UCP1 through the upregulation of the AMPK/PGC1α pathway. The increased level of UCP1 in the brown adipose tissue further initiates the generation of heat by releasing the stored chemical energy. Hence, here the stored fat is burned to release the energy [99]. Concurrently, mitochondrial transcription factor A and nuclear respiratory factor 2 will be upregulated to support the mitochondrial biogenesis process in assisting the breakdown of stored fats by producing enough heat [97]. Meanwhile, quercetin increases the level of UCP1 through the AMPK-Sirt1 signalling pathway. Quercetin inhibits adipocytes differentiation by upregulating the gene expression involved in brown-like adipocyte-specific genes, such as cell death-inducing DNA fragmentation factor alpha (DFFA)-like effector α (CIDEA), T-box transcription factor 1 (TBX1), and positive regulatory domain containing 16 (PRDM16) [100].

**Figure 3:**
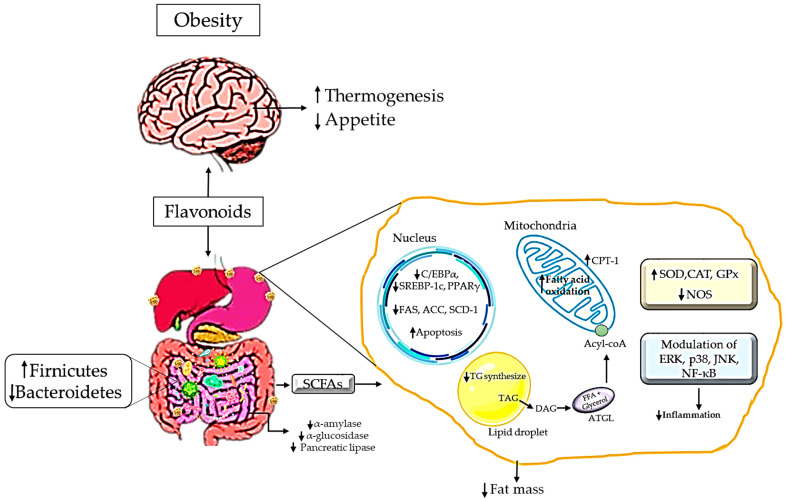
Mechanism of action of flavonoids in managing obesity.

In addition, flavonoids, such as Isoquercitrin and Isorhamnetin, are known for their ability to activate the Wnt/β-catenin signalling pathway [101]. The initiation of the Wnt/β-catenin pathway could inhibit adipogenesis and adipocytes differentiation via the action of β-catenin-dependent and -independent activity [102]. Another possible explanatory mechanism could be the inhibition of C⁄EBPα expression and PPARγ, which modulate the gene expression involved in adipogenesis [103]. In addition, quercetin, kaempferol, apigenin, rutin, and baicalein enhance the production of cholecystokinin (CCK) [104], thereby inhibiting excessive food intake [105], while epicatechin and anthocyanins stimulate the release of GLP-1 from the enteroendocrine cells in the intestine [106], thereby promoting satiety [107]. Figure 3 summarizes the mechanism of action of flavonoids as a natural antioxidant in managing obesity.

**Table 3:** In vivo evidence on flavonoids as an anti-obesity agent.

Author	Flavonoids	Animal	Dosage and Duration	Mode of Intervention	Findings
Lu et al., 2013 [108]	Citrange Fruit Extracts	6-week-old female C57BL/6 mice	1% *w*/*w* for 8 weeks	Diet	- Decreased body weight and food intake. - Decrease in fasting blood glucose level. - Decreased level of triglycerides, total cholesterol, and LDL in serum. - Decreased expression of PPARγ, adipocyte fatty-acid-binding protein, acetyl-CoA carboxylase, fatty acid synthase, liver X receptor β (LXRβ), lipoprotein lipase, and apolipoprotein E in the liver. - Increased expression of TP-binding cassette transporter G1 (ABCG1) in the liver.
Xu et al., 2013 [109]	Luteolin	5-week-old C57BL/6 mice	0.002% or 0.01% for 6 weeks	Diet	- Decreased level of body weight, leptin, and insulin. - Increased level of adiponectin in serum. - Increased number of apoptotic cells in the adipocytes. - Decreased level of F4/80, MCP-1, TNF-α, and IL-6 in the serum. - Decreased number of mast cells in the adipose tissue epididymis. - Inhibition of PKC translocation in the mast cells.
Dong et al., 2014 [110]	Quercetin	4-week-old C57BL/6 mice	0.1% *w*/*w* for 12 weeks	Diet	- Decreased level of body weight and adipose tissue weight. - Increased expression of GLUT4, phosphorylated Akt and LKB1 in the epididymis adipose tissue. - Decreased number of mast cell protease-4 and uncoupling protein 1, CDLLC, and NOS2 in the epididymis adipose tissue. - Increased level of MGL2 and CHIL3L in the epididymis adipose tissue. - Decreased level of TNF-α, IL-6, and MCP-1 in the epididymis adipose tissue. - Increased activity of AMPKα1 and SIRT1 in the epididymis adipose tissue.
Tan et al., 2014 [111]	Fortunella margarita fruit extract	12-week-old female C57BL/6 mice	1% *w*/*w* for 8 weeks	Diet	- Decreased level of fasting blood glucose, total cholesterol, triglycerides, and LDL. - Increased expression of PPARα, cluster of differentiation 36 (CD36), CYP4a10, CYP4a14, acyl-CoA oxidase (ACO) and stearoyl-CoA desaturase-1 (SCD1). - Increased level of uncoupling protein (UCP)-2 in the brown adipose tissue.
Assini et al., 2015 [112]	Naringenin	8–12 weeks of male Fgf21^−/−^ C57BL6/J mice	3% *w*/*w* for 4 weeks	Diet	- Decrease in the body weight. - Decreased of visceral adipose tissue volume. - Decreased percentage of adipocytes in the epididymal fat. - Decreased level of leptin, adiponectin, and TNFα in the plasma. - Increased level of PGC1A, CPT1A, and UCP-1 in the adipocytes. - Decreased level of fat accumulation in the liver.
Zang et al., 2015 [113]	Kaempferol glycosides	6-week-old male C57BL/6J mice	0.15% for 92 days	Diet	- Decrease in body weight. - Decreased adipose tissue weight in pararenal, epididymal, mesenteric, and visceral. - Decreased level of fasting blood glucose, leptin, insulin, and TNF-α in serum. - Decreased level of triglycerides in liver. - Decreased level of FAS, PPARγ, and SREBP-1 in the liver. - Increased level of CPT in the liver.
Bao et al., 2016 [114]	Decussatin	6-week-old male Wistar rats	20 mg/kg for 12 weeks	Diet	- Decreased body weight. - Decreased weight of liver, kidney and adipose tissues. - Decreased level of insulin, leptin, and NEFA in serum. - Decreased level of triglycerides, cholesterol, and LDL in serum. - Decreased level of FAS in the serum.
Pan et al., 2016 [115]	Lychee extracts, green tea polyphenols, and citrus polymethoxyflavones	4-week-old male C57BL/6J mice	0.1 or 0.5% for 16 weeks	Diet	- Decrease in body weight. - Decrease in weight of perigonadal, retroperitoneal, and mesenteric fat. - Decrease in adipocytes size. - Decreased level of triglycerides, and total cholesterol in serum. - Decreased level of PPARγ, SREBP-1c, FAS, and ACC in the adipose tissue. - Increased level of Dlk1/Pref-1 in epididymal adipose tissue. - Increased phosphorylation of AMPK in the adipose tissue.
Ke et al., 2017 [116]	Naringenin	20-week-old C57BL/6J mice	1 or 3% for 13 days	Diet	- Decrease in body weight and calorie intake. - Decreased level of blood glucose. - Decrease in mammary, perigonadal, and mesenteric adipose tissue. - Decreased level of MCP-1, IL-6, and leptin in the adipose tissue.
Kwon et al., 2017 [117]	Flavonoid glycosides extract from Seabuckthorn leaves	4-week-old male C57BL/6J mice	0.04% for 12 weeks	Diet	- Decreased level of body weight. - Decreased weight of white adipose tissue, epididymal, perirenal, mesenteric, subcutaneous, and interscapular fats. - Decreased level of triglycerides, total cholesterol, FFA, apolipoprotein B, and atherogenic index in the plasma. - Decreased level of FAS, ME, PAP, ACAT, and SREBP-1c in the plasma. - Increased level of CPT1α in the plasma.
Burke et al., 2018 [118]	Naringenin, Nobiletin	10–12-week-old Ldlr^−/−^ mice	0.3 or 3% for 12 weeks	Diet	- Decrease in body weight. - Decrease in epididymal, visceral, and subcutaneous fat mass. - Decreased level of TNFα, CCL2, and CCL3 in the adipose tissue. - Increased level of UCP1 in the epididymal and inguinal adipose tissue. - Decreased level of insulin, LDL, total cholesterol, and triglycerides in the plasma. - Decreased level of SREBP-1c, CCL2, CCL3, TNFα, and IL-1β in the liver. - Increased level of CPT1A and PGC1α in the liver.
Sheng et al., 2019 [119]	Mulberry leaves	4-week-old C57BL/6J mice	20% for 13 weeks	Diet	- Decreased level of fasting blood glucose and plasma insulin. - Decreased level of ALT, AST, ALP, and creatinine in the serum. - Increased level of energy expenditure. - Increased level of f UCP1, PGC-1α, PGC-1β, CPTα, CEBP/α, and CEBP/β in brown adipose tissue. - Decreased level of PPARγ in the brown adipose tissue. - Decreased level of *Firmicutes*, and *Proteobacteria* colonization in the gut. - Increased level of *Bacteroidetes*, and *Verrucomicrobia* colonization in the gut.
Bian et al., 2021 [120]	Kaempferol	8-week-old male C57BL/6J mice	0.1% for 16 weeks	Diet	- Decrease in body weight, and adipocytes size. - Decreased level of total cholesterol, and triglycerides in serum. - Decreased level of plasma glucose, IL-6, TNF- α, IL-1 β, and MCP-1 in the adipose tissue. - Decreased level of CD11b^+^/F4/80^+^ in the colon tissue. - Increased expression of ZO-1, occludin and claudin-1 in the colon tissue. - Decreased level of TLR4, MyD88, p65 and translation of p65 in the colon tissue. - Decreased level of *Firmicutes*, and *Proteobacteria* colonization in the gut. - Increased level of *Bacteroidetes*, and *Verrucomicrobia* colonization in the gut.
Miranda et al., 2016 [121]	Xanthohumol	8-week-old male C57BL/6J mice,	0.033 or 0.066% for 12 weeks	Pellet	- Decreased level of body weight. - Decreased level of LDL, IL-6, MCP-1, insulin, and leptin in plasma. - Increased level of HDL in the plasma. - Decreased level of triglycerides, and PCSK9 in the plasma and liver.
Nascimento et al., 2013 [122]	Camu–camu pulp	Male wistar rats	25 mL for 12 weeks	Ingestion	- Decrease in weight of visceral and epididymal tissue. - Decreased level of triglycerides, cholesterol, LDL, and VLDL in the serum. - Decreased level of AST, ALT and ALP. - Decreased level of TNF-α in the serum.
Alam et al., 2013 [123]	Naringin	9–10-week-old male wistar rats	100 mg/kg/day for 8 weeks	Oral gavage	- Decreased level of retroperitoneal fat deposition in the abdomen. - Decreased level of total cholesterol, triglyceride, non-essential fatty acid, insulin, and glucose in the plasma.
You et al., 2013 [124]	Nelumbo nucifera	4-week-old Sprague Dawley rats	400 mg kg/day for 7 weeks	Oral gavage	- Decreased level of body weight, and food efficacy. - Decreased weight of adipose tissue. - Decreased level of total cholesterol, LDL, and insulin.
Yoshida et al., 2014 [125]	Naringenin	7-week-old male C57BL/6J mice	100 mg/kg/day for 1, 7, and 14 days	Oral gavage	- No effect on body weight. - Decreased level of Mac-2, and MCP-1 in the adipocytes. - Decreased level of phosphorylated JNK in the adipocytes.
Baselga-Escudero et al., 2015 [126]	Proanthocyanidins	6-week-old female Wistar rats	5, 25, or 50 mg/kg for 3 weeks	Oral gavage	- Decreased level of body weight. - Decreased level of triglycerides, total cholesterol, and total lipid in the liver. - Decreased level of ABCA1, IRS2, fatty acid synthase (FAS), and Akt3 in the liver.
Miyata et al., 2015 [127]	Xanthohumol	6-week-old male C57BL/6J mice	75 or 150 mg/kg/day for 3 days	Oral gavage	- Decreased level of body weight. - Decreased weight of liver, epididymal, mesenteric, sub-cutaneous, inguinal white fat pads, interscapular brown fat pads and fat in abdomen and liver. - Decreased expression of SREBP-1 in the liver. - Decrease in subcutaneous and abdomen fat volume. - Decreased level of lipid absorption in the small intestine. - Decreased level of cholesterol, triglycerides, and LDL. - Decreased level SREBP-1, ACC1, FAS, and SCD1 in the liver. - Increased level of ATGL, adiponectin, and UCP1 in the liver.
Qin et al., 2015 [128]	3-O-[(E)-4-(4-cyanophenyl)-2-oxobut-3-en-1-yl] kaempferol	4-week-old Male C57BL/6 mice	2, 5 or 50 mg/kg for 4 weeks	Oral gavage	- Decrease in body weight. - Decrease in visceral fat pad in the epididymis, mesenteric, and perirenal adipose. - Decreased level of blood glucose, leptin, cholesterol, triglycerides, nonesterified fatty acids, and LDL in the serum. - Increased level of adiponectin in the serum. - Decreased level of TNF-α, IL-1β, MCP-1, and MDA in the serum. - Increased level of serum superoxide dismutase (SOD). - Decreased level of fat accumulation and adipocytes size in the liver. - Decreased level of FAS, sterol regulatory element-binding protein 1c (SREBP-1c), CCAAT/enhancer-binding protein α (C⁄EBP α), peroxisome proliferators-activated receptors γ (PPARγ) lipolytic gene in the epididymal adipose tissues and liver. - Increased level of hormone-sensitive lipase (HSL) in the in the epididymal adipose tissues and liver. - Decreased level of HIF-1α, phosphoenolpyr- uvate carboxykinase (PEPCK), and glucose-6-phosphatase (G6P) in the liver. - Increased phosphorylations of AMPK and ACC in the liver. - Decreased level of S6K1 mRNA in the adipose tissue and muscle.
Liu et al., 2017 [129]	Flavonoid rich extract of paulownia fortunei flowers	Male ICR mice	50 and 100 mg/kg for 8 weeks	Oral gavage	- Decrease in body weight and calorie intake. - Decreased level of ALT, AST, glucose, insulin, HOMA-IR, total cholesterol, triglycerides, and LDL in serum. - Increased level of HDL in the serum. - Increased of AMPK phosphorylation in the liver. - Decreased level of SREBP-1c, 3-hydroxy-3-methylglutaryl-CoA reductase (HMGCR), IRS-1, and FAS in the liver. - Increased level of carnitine palmitoyltransferase 1 (CPT1) in the liver.
Gentile et al., 2018 [130]	Apigenin	6-week-old male C57BL/6 mice	1, 10 or 30 mg/kg/day for 8 weeks	Oral gavage	- Decrease in body weight and epididymal fat weight. - Decrease in total cholesterol, triglycerides, and glucose in blood. - Decreased level of MDA, IL-1β and IL-6 in the colon tissue. - Decreased level of eosinophil infiltration in the colon tissue. - Decreased level of iNOS in the colonic myenteric ganglia.
Bai et al., 2019 [131]	Quzhou Fructus Aurantii extract	8-week-old male C57BL/6J mice	300 mg/kg for 12 weeks	Oral gavage	- Decrease in body weight. - Decreased level of total cholesterol, triglycerides, NEFA, LDL, and fasting blood glucose. - Absence of hepatocytes ballooning, and infiltration of inflammatory cells in the liver. - Increased expression of CLDN3 and OCLN in the colon tissue. - Suppression of NF-κB p65 and IKKα/β phosphorylation in the colon.
Feng et al., 2016 [67]	Apigenin	3 to 4-week-old male C57BL/6J mice	10, 30, or 50 mg/kg for 21 days	Intraperitoneal Injection	- No effect on body weight. - Decreased level of IL-12, TNF-α, IL-6, and IL-1β in the adipose tissue. - Decreased level of CCL2, MHCII, and CD80, CCL3, and CCL4 in the macrophages. - Increased level of IL-10, CD206, and Fizz1 in the macrophages. - Decreased expression of PPARγ. - Decreased level of ALT, AST, total cholesterol, and triglycerides.
Varshney et al., 2019 [132]	Quercetin, rutin, kaempferol, and myricetin	7-week-old male C57BL/6 mice	25 mg/kg/day for 4 weeks	Intraperitoneal injection	- Decreased level of body weight. - Decreased level of triglycerides, total cholesterol, NEFA, fasting blood glucose, and insulin.
Romero-Juárez et al., 2021 [133]	Kaempferol	8-week-old C57Bl/6J male mice	0.5 mg/kg/day for 40 days	Intraperitoneal injection	- Decrease in body weight and feed efficacy. - Decreased level of blood glucose. - Decrease in density and activation levels of microglia in the arcuate nucleus (ARC).
Yang et al., 2016 [134]	Flavonoid-enriched extract from Hippophae rhamnoides	8-week-old C57BL/6 mice	100 or 300 mg/kg for 9 weeks	Intragastric administration	- Decreased level of body weight. - Decreased level of triglycerides, and total cholesterol in the serum and the liver. - Decreased level of fasting blood glucose, and OGTT. - Decreased level of adipocytes size, and epididymal fat pad in the liver. - Decreased level of PPARγ, and TNFα in the liver. - Increased level of PPARα, CCAAT/enhancer-binding protein a (C/ EBPα) in the liver.
Dai et al., 2018 [135]	Baicalin	Mice	400 mg/kg/day for 12 weeks	Intragastric administration	- Decreased level of body weight, percentage of body fat, liver weight, and hepatic fat accumulation. - Decreased level of total cholesterol, triglycerides, and NEFA in the liver. - Increased level of CPT1A, and total ketone bodies in the plasma. - Increased level of energy expenditure.
Shen et al., 2019 [136]	Neohesperidin, hesperidin, and naringin	4-week-old male C57BL/6 mice	50, 100, or 200 mg/kg/ day for 12 weeks	Intragastric gavage	- Decrease in body weight. - Decreased level of triglycerides, total cholesterol, LDL, leptin, and LPS in the plasma. - Decreased of liver weight, adipocytes size, and absence of infiltration of inflammation in the liver. - Increased level of SOD, and CAT in the liver. - Decreased expression of PPARγ, C/EBPα, FAS, and ACOX1 in the liver. - Increased level of *Bifidobacterium*, *Pikenella*, *Musicpirilum*, *Anaeroplasma*, *Odoribacter*, and *Eubacterium* in the gut.
Su et al., 2019 [137]	Apigenin	C57BL/6 male mice	15 or 30 mg/kg/day for 13 days	Subcutaneous injection	- Decrease in body weight and size of visceral adipocytes. - Decreased phosphorylation of STAT3 in the visceral adipose tissue. - Decreased level of CD36, and PPARγ in the adipocytes.

## 6. Flavonoids in the Modulation of Gut Microbiota in Obesity

Flavonoids play an important role in the modulation of the gut microbiome in exerting its anti-obesity effects, particularly in protecting against obesity-induced inflammation. Gut dysbiosis is one of the common features seen in obesity. In HFD-induced obese rodent models, a high level of *Proteobacteria* with a reduced level of *Patescibacteria* at the phylum and a decreased level of *Muribaculaceae* and increased level of *Bacteroidaceae*, *Tanerellaceae*, *Desulfovibrionacaeae*, and *Lachnospiraceae* at the family level are commonly observed [138]. Similarly, another study reported that a decreased level of genus *Bacteroidetes* and increased genus *Firmicutes* microbes, such as *Clostridium*, *Lactobacillus*, and *Ruminococcus*, with a decreased level of *Firmicutes* classified microbes, such as *Faecalibacterium prausnitzii*, are observed in obesity [139]. Such changes in the gut microbial composition may contribute to an increase in body weight by inducing changes in the intestinal structure. Some of the notable changes include the translocation of intestinal protein, an increase in pro-inflammatory factors and gut permeability [138], a reduction in mucus production, and the suppression of cystic fibrosis transmembrane receptor gene at the ileal enterocytes [139], which may eventually lead to “leaky gut” [138]. In such circumstances, the disruption in the intestinal barriers fails to prevent the entrance of harmful bacterial metabolites or endotoxin from the gut lumen into the circulation, which may trigger the activation of inflammatory response [140]. Specifically, a high level of IL-1β impairs insulin signalling in the macrophages and peripheral tissues [141], which will in turn contribute to obesity development, as a slight elevation of insulin in the circulation can effectively inhibit lipolysis and enhance lipogenesis in the adipocytes [142].

However, flavonoids are able to stabilize the microbiome ecosystem in the gut via various pathways. For this, a high content of epicatechin, catechin, gallic acid, gallic acid, and caffeic acid in tea extract successfully stimulated the growth of commensal bacterial populations such as *Clostridium* spp., *Bifidobacterium* spp., and *Lactobacillus* spp. in the gut. At the same time, 0.1% *w*/*v* of tea extract inhibited the growth of pathogenic microbes, including *Bacteroides* spp., *Clostridium perfringens*, and *Clostridium difficile*, in the intestine [143]. Similarly, the high concentration of anthocyanins and proanthocyanidins from grapes successfully reduced fat deposition in the inguinal, retroperitoneal, epididymal, and mesenteric white adipose tissue by promoting the growth of microbes from the genus *Coprococcus* and repressing the growth of microbes from the genus *Ruminococcus* [144]. Similarly, the combination of quercetin and resveratrol at the dose of 30 mg/kg/day for 8 weeks successfully constrained the growth of genus *Firmicutes* and *Lachnoclostridium* in HFD-induced wistar rats. As a result, the level of lipids, leptin, and adipose tissue mass were suppressed [145].

A contemporary investigation has shown that lactic acid bacteria, such as *Lactobacillus,* are able to restore lipid and cholesterol metabolism, thereby preventing excessive weight gain [146], and quercetin and resveratrol are effective in improving the population of Lactobacillus in the gut [145]. Analogously, 15 mg/kg of trans-resveratrol diet is able to upregulate the expression of tight junction proteins and occludin in the colon via the regulation of the gut microbiome. In such conditions, the barrier integrity in high fat sucrose-induced obese rats was restored within 6 weeks [147]. In a similar fashion, Noratto et al. (2014) reported that the high flavonoid content in *Prunus persica* and *Prunus salicina* fruit exerts an anti-obesity property by favouring the microenvironment for *Faecalibacterium*, *Lactobacillus*, and *Bacteroidetes*, ergo reducing the faecal short chain fatty acids [148]. Meanwhile, isoflavones from soy bar stimulate lipid catabolism by increasing the beneficial microbial populations (*Bifidobacterium* spp.) in the gut [149]. In 2018, Gentile and colleagues reported that the oral administration of 10 mg/kg/day of Apigenin for 8 weeks is sufficient to reduce body weight and epididymal fat mass, as well as serum triglycerides and cholesterol level [80]. One of the plausible explanations for this could be the ability of Apigenin to modulate the gut microbial composition at a very minimum dosage [150].

## 7. Flavonoids in Redox Modulation in Obesity

Redox balance is essential in ensuring biological processes under optimum conditions, and redox imbalance will interrupt the mitochondrial functions, intestinal microbial composition, and ectopic lipid deposition [151]. In a normal state, mitochondria are responsible for the intracellular formation of reactive oxygen species (ROS). However, in obesity, chronic low grade inflammatory conditions cause the systemic oxidative stress to increase due to the electron leakage at complex III. This may eventually result in an excessive amount of superoxide generation. Similarly, the high concentration of free fatty acids in the plasma and storage of fat in the white adipose tissue exceeding its capacity may induce the generation of superoxide radicals in the mitochondrial electron transport chain [152]. These superoxide radicals, also known as prime ROS, are mainly generated by the mitochondrial carriers via the oxidation of hydrogen peroxide. Here, the SOD act as the catalysing enzymes. The ROS formed will then be neutralized by SOD within the mitochondria, while only a very minimal concentration of ROS will enter the cytosol [153]. But, in obesity, the deposition of high amounts of adipose tissue will raise the formation of pro-inflammatory cytokines, causing the level of ROS to increase [154]. When the ROS level exceeds the normal level, continuous ROS generation via mitochondria and other sources will continue, which may hinder the normal activity of the respiratory chain and Krebs cycle enzymes, thereby leading to mitochondrial dysfunction, and ultimately lead to the development of obesity [153]. Simultaneously, the excessive level of ROS will suppress the activity of antioxidant enzymes, such as SOD, catalase (CAT), and glutathione peroxidase (GPx) in the white adipose tissue [155]. Hence, a high level of fat accumulation, as seen in obesity, is often associated with redox imbalance [156], causing cellular dysfunction by stimulating the redox-sensitive signalling pathway and its downstream modulators [157].

Furthermore, flavonoids intake has been proven to reverse redox imbalance in obese rodents. To support this statement, Macedo et al., in 2017, conducted an in vivo trial to assess the effect of green tea extract on obese male wistar rat’s brain tissue. The outcome of the study showed that the oral administration of green tea extract for 12 weeks successfully restored the reduced levels of MnSOD, CuZnSOD, CAT, glutathione reductase, and total antioxidant capacity (TAC) in the cerebral cortex of the obese rats [158]. Similarly, the oral administration of 10 mg/kg of fisetin for 12 weeks in male albino rats successfully mitigated oxidative stress by downregulating hepatic lipid peroxidation via the normalization of the thiobarbituric acid reactive substances (TBARS) level and increasing the expression of TAC in the obese rat’s liver. The amelioration of the antioxidant status in the obese rat’s liver in due course resulted in the suppression of thioredoxin-interacting protein and PARP activation, thus balancing the redox status [159]. Similarly, Zeng et al. (2017) observed the same feature in the obese mice treated with Dihydromyricetin. The observation showed that flavonoid supplementation could improve the mitochondrial respiratory activity by upregulating the Sirtuin-3 dependent signalling mechanism. As a result, the mitochondrial antioxidant enzyme activities, such as SOD2, were raised through the deacetylation of protein/lysine. This was further proven by the decreased level of TBARS and 4-hydroxynonenal in the obese mice’s liver, indicating the suppressed oxidative stress level [160]. In short, flavonoid compounds have been identified as potential targets for preventing obesity due to their ability to reduce adipose tissue mass, which in turn decreases the formation of intracellular free radicals, enhances endogenous antioxidants, and reduces inflammatory signalling pathways. Animal studies have provided evidence for the efficacy of flavonoids in this regard, highlighting their usefulness in suppressing oxidative stress and the related inflammatory conditions associated with obesity.

## 8. Bioavailability of Flavonoids

The effectiveness and therapeutic potential of flavonoids are limited by their low and poor bioavailability, which may significantly influence the impact of their nutritional effects. Bioavailability is best known as the rate and degree of the proportion of a nutrient or active substance that are absorbed and made available for tissue distribution, where they can have physiological effects after ingestion. According to Manach et al. (2005), the best rate of absorption among flavonoids is seen in gallic acid and isoflavones, followed by catechins, flavanols, flavanones, flavonols, anthocyanins, and galloylcatechins [161]. According to Akhlaghi and Foshati (2017), despite having strong absorption, gallic acid conjugation with catechins reduces the bioavailability of catechins [162]. The bioavailability of flavonoids varies between subclasses of flavonoids and individual compounds of flavonoids. Many factors have been proposed to contribute to the varying low bioavailability of flavonoids, namely the subclass, molecular weight, composition, esterification, and glycosylation [163]. Recent research has revealed that the gut microbiota also plays a critical role in the metabolism and absorption of flavonoids, which can greatly impact their bioavailability. A polyphenol can be changed by the gut microbiota through enzymatic processes such as fermentation, oxidation, reduction, and hydrolysis. The chemical structure of these substances can change as a result of these biotransformations, which may impact their solubility, stability, and absorption [164].

Additionally, the food matrix and processing techniques can also impact the bioavailability of flavonoids [165], while complex structures of flavonoids and higher molecular weights result in poorer bioavailability [166]. Flavonoids are typically found as glycosides that are conjugated to sugars compared to their aglycone form. Flavonoids can also occur as methylated derivatives. Glycosylation makes a substance more water-soluble and increases its stability in water, which promotes its absorption from the digestive tract and increases its bioavailability [162,167]. D-glucose, galactose, L-rhamnose, arabinose, or glucorhamnose are the common carbohydrates that are bonded to flavonoids via glycosidic linkage at position 3 or 7 [168]. In contrast, according to Naeem et al. (2021), despite the fact that most flavonoids occur in glycosidic form, aglycones have better bioavailability and faster absorption than glycosides due to their superior membrane interactions. Additionally, free hydroxyl groups in flavonoids have been linked to reducing oral bioavailability because they quickly conjugate through glucuronidation and sulfation. Flavanoids are liberated from their glycosidic linkages and go through a variety of chemical changes during digestion, including methylation and sulfation, which can have an impact on their bioavailability [163].

## 9. Conclusions

In conclusion, this review provides in vivo evidence supporting the potential protective role of flavonoids as natural antioxidants in managing obesity. Flavonoids offer an alternative approach to traditional obesity management, which often relies on pharmaceutical drugs and surgeries that have unfavourable side effects. The review provides an overview of the in vivo evidence on flavonoids as anti-obesity agents that have shown promising outcomes. Additionally, the review highlights the mechanisms of action by which flavonoids act as natural antioxidants in managing obesity, including their modulation of gut microbiota and redox balance. However, further research is necessary to fully understand the effectiveness and safety of flavonoids as an anti-obesity intervention, particularly in the long-term. Future studies should also investigate the translation of preclinical findings to the clinical setting, appropriate dosages and guidelines, and elucidate the molecular mechanisms responsible for the beneficial effects of flavonoids. Ultimately, this review suggests that flavonoids offer a promising avenue for the management of obesity and its related co-morbidities.

## Figures and Tables

**Table 1:** Current available intervention for managing obesity and its side effects.

Pharmacotherapy	
Types	Side Effects
Liraglutide (Saxenda^®^) [32]	Abdominal pain, constipation, vomiting, dyspepsia, fatigue, dizziness
Orlistat [33]	Steatorrhoea, bloating, oily spot, fecal urgency, fecal incontinence
Naltrexone/bupropion (Mysimba^®^) [34]	Increased risk of epileptic fits, nausea
Lorcaserin (Belviq^®^) [35]	Mitral regurgitation, depression, pulmonary hypertension
Phentermine/topiramate (Qsymia™) [36]	Paresthesia, dry mouth, dysgeusia, insomnia, dizziness, constipation
Oxyntomodulin [37]	Nausea, vomiting, diarrhea, acute pancreatitis, decreased blood pressure, increased risk of certain types of cancer
Beloranib [38]	Insomnia, decreased of mean systolic blood pressure
Surgical intervention	
Bariatric surgery [39]	Loss of lean body mass and muscle, micronutrient deficiency
Laparoscopic adjustable gastric banding [40]	Pouch enlargement, band slip, band erosion, intra-abdominal infection, port breakage
Roux-en-Y gastric bypass [41]	Stenosis at gastrojejunostomy, internal hernia, gallstones
Sleeve gastrectomy [42]	Midstomach stenosis, weight re-gain
Biliopancreatic diversion with a duodenal switch [43]	Bone pain, diarrhea, vomiting
Non-surgical intervention	
Intragastric balloon [44]	Vomiting, hypokalemia, renal insufficiency, abdominal pain, gastroesophageal reflux
